# Fluorescein Derivatives in Intravital Fluorescence Imaging

**DOI:** 10.3390/cells2030591

**Published:** 2013-08-02

**Authors:** Thomas A. Robertson, Florestan Bunel, Michael S. Roberts

**Affiliations:** 1 Therapeutics Research Centre, School of Pharmacy and Medical Sciences, Division of Health Sciences, University of South Australia and Basil Hetzel Institute for Medical Research, GPO Box 2471, Adelaide, SA, 5001, Australia; 2 Université Libre de Bruxelles, Campus Plaine CP205, Boulevard du Triomphe, 1050 Bruxelles, Belgium; 3 Therapeutics Research Centre, School of Medicine, The University of Queensland, Princess Alexandra Hospital, Woolloongabba, QLD, 4102, Australia

**Keywords:** intravital, FLIM, fluorescein

## Abstract

Intravital fluorescence microscopy enables the direct imaging of fluorophores *in vivo* and advanced techniques such as fluorescence lifetime imaging (FLIM) enable the simultaneous detection of multiple fluorophores. Consequently, it is now possible to record distribution and metabolism of a chemical *in vivo* and to optimise the delivery of fluorophores *in vivo*. Recent clinical applications with fluorescein and other intravital fluorescent stains have occurred in neurosurgery, dermatology [including photodynamic therapy (PDT)] and endomicroscopy. Potential uses have been identified in periodontal disease, skin graft and cancer surgery. Animal studies have demonstrated that diseased tissue can be specifically stained with fluorophore conjugates. This review focuses on the fluorescein derived fluorophores in common clinical use and provides examples of novel applications from studies in tissue samples.

## 1. Introduction

Drug and chemical absorption and disposition *in vivo* have commonly been characterised indirectly with analytical instrumentation using drug quantitation following tissue sampling. Intravital fluorescence microscopy enables the direct imaging of fluorophores *in vivo* and fluorescence lifetime imaging (FLIM) enables the simultaneous analysis of multiple endogenous and exogenous fluorophores with identical emission wavelengths. Pharmaceutical active ingredients ideally target specific regions of tissue and imaging techniques can be used to characterise the disposition of a single component within a mixture. For example, the benefits of sunscreen (zinc oxide, titanium dioxide) are obtained at the skin surface and undesirable effects could potentially occur if the sunscreen is absorbed [1]. Small molecule drugs are formulated to be well absorbed and sometimes have systemic effects which can be observed through imaging.

Fluorescence confocal microscopy has advantages over reflectance microscopy. Noise is minimised as filters are used to distinguish the emitted fluorescent signal from the excitation source. Multiphoton microscopy has the advantage of a smaller focal volume than single photon confocal microscopy. Near infra-red (nIR) light excitation limits phototoxicity and provides deeper penetration than visible light into live tissue. FLIM can be used to measure endogenous fluorophores as well as separating the signals from multiple fluorophores. 

Disadvantages which may limit clinical uses of fluorescence microscopy are the relatively small number of fluorescent stains approved for use in humans and the requirement for additional specialised equipment compared with reflectance imaging. The fluorescent dyes in widespread clinical use as injected imaging agents are fluorescein, methylene blue, indocyanine green and the prodrug5-aminolevulinate (5ALA) [2,3]. Other dyes in common use as topical or gastrointestinal contrast agents include acridine orange and the structural derivative acriflavine. In addition to 5ALA, which is a prodrug of protoporphyrin IX (PPIX), a range of photosensitizers are in clinical use or development and are potentially useful in applications with fluorescence imaging. Recent clinical applications with intravital fluorescent stains have occurred in neurosurgery, dermatology [including photodynamic therapy (PDT)] and endomicroscopy. Potential uses have been identified in periodontal disease [4], skin graft and cancer surgery. Animal studies have demonstrated that diseased tissue can be specifically stained with fluorophore conjugates as small molecules such as a fluorescein folate conjugate (see Section 6) or as macromolecules, such as fluorescein dextran and rhodamine B dextran conjugates (see Section 7). In this review, we aim to provide a summary of the physicochemical properties of fluorophores which are structurally related to fluorescein and used either *in vivo* in humans or are relevant for comparison as histology stains. We conclude with a discussion of some existing clinical applications with intravital imaging using endogenous fluorescence or fluorescein stain.

## 2. Endogenous Fluorescence

Live tissue is fluorescent and the endogenous signal may interfere with exogenous fluorescence resulting from an applied dye. The spectra due to endogenous tissue fluorescence in living tissue can be used to identify changes in cell physiology and to diagnose disease [5,6]. This dynamic fluorescence signal is a composite due to several endogenous molecules, is altered in chemically fixed tissue and rapidly changes when tissue is excised. As described in Table 1, ultra-violet (UV) and blue light stimulate fluorescence of several molecules including proteins, porphyrins and small molecules. The most intense endogenous fluorescence signal results from flavine adenine dinucleotide (FAD) and nicotinamide adenine dinucleotide phosphate [NAD(P)H]. The fluorescent component of FAD, riboflavin is in development for use to treat keratoconus by corneal collagen crosslinking [7,8,9].

**Table 1:** Fluorescence properties of endogenous molecules.

Endogenous fluorophore	Excitation	Emission	Fluorescence lifetime τ (ns) [10]
Elastin	415	475–575	0.2–2.5
FAD	~450	525–550	5.2
Keratin	277	450–500	1.6
NAD(P)H	360	450–460	0.3/2.3
PPIX [3]	~400	635	11
Retinol	351	~500	-

Autofluorescence has been used to diagnose colorectal cancer [11] and lung cancer [12]. The two channel system (λ_ex_ 390–470 nm, λ_ex_ 540–560 nm, Olympus AFI) detects changes in endogenous fluorophore intensity associated with diseased tissue.

High levels of an endogenous molecule dosed as a drug result in elevated levels in tissue and aid in margin mapping during surgery. For example, administration of precursors of PPIX such as 5-aminolevulinic acid and methyl 5-aminolevulinate result in elevated levels of PPIX in tissue [13]. During PDT, laser light is absorbed by PPIX and induces tissue necrosis. Use of PPIX is documented in glioma surgery [14], urology [3] and dermatology. Fluorescence-guided surgery with 5ALA (Gliolan) reportedly results in a twofold increase in the number of patients free from residual malignant glioma [14]. A synthetic analogue of PPIX, Meta-tetrahydroxyphenylchlorin (mTHPC, Tempoforin or Foscan) has been developed for the treatment of head and neck squamous cell carcinoma cancer *via* photodynamic therapy [15]. Use of FLIM in image guided brain surgery demonstrated spectroscopic differences in unstained tissue such that glioblastoma multiforme (GBM) tissue has an increased fluorescence lifetime (τ~1.6 ns) compared with healthy tissue (τ~1.3 ns) [16]. Changes in endogenous tissue fluorescence have been identified in biopsies from patients with cervical cancer [17] and contrast agents including fluorescein and dye conjugates have also been studied.

## 3. Common Small Molecule Fluorophores

Traditional methods in histopathology involve fixing, sectioning and staining tissue. For example, hematoxylin and eosin (H&E) stain is a popular method in medical diagnosis of disease. Hematoxylin stains cell nuclei blue and eosin counterstains cytosol and intercellular proteins red to pink. Hence, it is well established that a combination of dyes provides superior contrast between tissue components. 

Many dyes are used to stain tissue samples with a subset suitable for intravital studies in animals and cultured cells. A small number of fluorescent dyes are regulator approved for use in humans. In addition, some food colours and a small number of drugs are incidentally fluorescent and approved for use in humans. Whilst superior stains have been commercialised for cultured cells and animal studies, this article emphasises fluorophores with potential for use in intravital human studies and focuses on derivatives of fluorescein.

## 4. Historical Perspective: the Origin of Fluorescein

**Figure 1:**
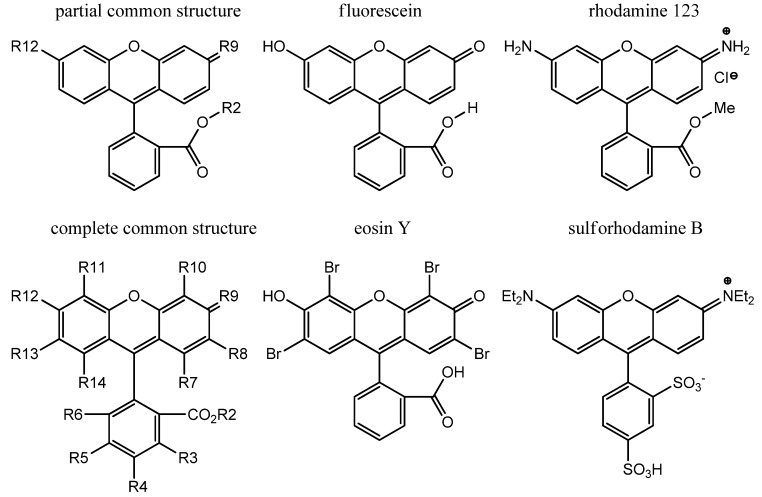
Common structures beside the structures of fluorescein, rhodamine, eosin Y and sulforhodamine B.

**Scheme 1:**
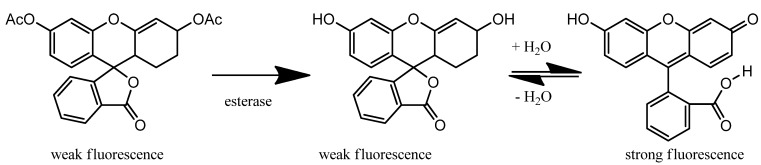
Metabolism of non-fluorescent FDA to fluorescein lactone and acid.

Physicochemical properties determine the suitability of small molecules for *in vivo* studies and especially, tissue penetration. Fluorescein is a commonly used example of an anionic fluorophore with uses in health care (e.g., ophthalmology, gastrointestinal endoscopy). Shown in Figure 1 and Table 2, fluorescein (R2=H, R12=OH, R9=O), rhodamine 123 (R2=Me, R12=NH2, R9=NH_2_^+^) and many derivative structures share the same tetracyclic core, can be synthesized by adduct formation with phthalic anhydride, and have been extensively studied since the first reported synthesis of fluorescein in the 1870s [18]. With a relatively long history, fluorescein acid and sodium salts have at least 18 and 73 names, respectively, such as 2-(6'-hydroxy-3'-oxo-3H-xanthen-9'-yl)-benzoic acid and uranin [19]. Shown in the complete common structure in Figure 1, the R-group numbering is in sequence with the carbon numbering in the systematic name (*i.e.*, R3 at carbon-3, R7 at carbon-1'). Many derivatives of fluorescein and rhodamine have been reported with intravital applications as dyes, fluorescent stains, food colourings and drugs. As shown in Scheme 1, fluorescein diacetate (FDA) is a non-fluorescent precursor to fluorescein that is enzymatically transformed to fluorescein lactone and fluorescein *in vivo*. FDA and derivatives, such as 6-carboxyFDA (6FDA), are used in various applications to assess cell viability in cell culture, microbe activity in soil samples, and intravital imaging of hepatic active transport [20]. When dissolved in aqueous media, fluorescein derivatives coexist as a mixture of fluorescent π-conjugated acid and non-fluorescent unconjugated lactone in pH dependent dynamic equilibrium [21]. The structures are used interchangeably in publications. 

**Table 2:** Functional groups present in the common structure.

Name	R2	R9	R12
fluorescein acid	H	O	OH
fluorescein sodium	Na	O	ONa
rhodamine 123	Me	NH_2_^+^	NH_2_

## 5. Synthesis

**Scheme 2:**
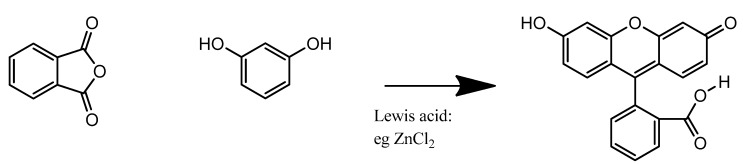
Fluorescein synthesis.

Shown in Scheme 2, fluorescein is commonly synthesised by Friedel-Crafts acylation from phthalic anhydric and resorcinol in a 1–2 molar ratio with zinc(II) chloride acting as a Lewis acid catalyst. Replacing resorcinol by N,N-diethylaminophenol in this reaction results in formation of rhodamine B. Hence, analogous synthetic procedures have been used to prepare a variety of fluorophores and dyes.

## 6. Eosinophilic / Anionic Stains

As discussed earlier, eosin is a popular confocal microscopy visible stain used in histopathology within the H&E stain. Whilst all forms of eosin are fluorescent structural derivative of fluorescein, Eosin Y is more commonly used. Eosin Y (Y = yellow, shown in Figure 1) and eosin B (B = blue) have similar structures, staining and spectral properties as shown in Table 3, Table 4 [22]. Eosin Y has been compared with reflectance H&E stain using fluorescence confocal microscopy [23,24]. Within the fluorescein series, it may be possible to identify a more fluorescent eosin Y substitute stain for use *in vivo* in humans. Fluorescein is a commonly used example of an anionic fluorophore with uses in health care [25], including ophthalmology and gastrointestinal endoscopy following topical and intravenous administration, respectively. The fluorescein and rhodamine series preferentially stain the cytosol and cell membrane.

All dyes have high visible extinction coefficients (ε, M^−1^cm^−1^) indicating that they absorb visible light at relatively low concentrations. Fluorescence quantum yield (Φ_F_) is a property which is indicative of the minimum concentration required for fluorescence detection and the relative concentration suitable within a mixture of fluorophores. Φ_F_ is the number of photons emitted per photons absorbed and is dependent on molecular structure and solvent. For example, in the series of fluorescein derivatives shown in Table 5, Table 6, the reported quantum yield varies significantly. When Φ_F_ is relatively large (arbitrary threshold > 0.1), significant fluorescence is suitable for tissue staining. For this series, the effect of additional electron withdrawing halogen groups is to red shift the absorption peak and lower Φ_F_ [26]. In contrast, shown in Table 4, Table 6, the addition of ether and ester groups to fluorescein retains high Φ_F_ [27]. 

Molecular binding is known to change spectral properties of fluorescein derivatives. For example, Zinpyr-1-4 is a series of R10-, R11-alkylated fluorescein derivatives in which Φ_F_ is always high but also enhanced in the presence of ionic zinc [32,33]. Consequently, Zinpyr-1 is a useful intravital stain for detecting organelle specific localisation of ionic zinc.

**Table 3:** R-groups within a series of fluorescein derivatives commonly used clinically in humans.

Name	R2	R5	R6	R8=R13	R9	R10=R11	R12
fluorescein	H	H	H	H	O	H	OH
eosin Y	H	H	H	Br	O	Br	OH
eosin B	H	H	H	NO_2_	O	Br	OH
carboxyfluorescein	H	CO_2_H	H	H	O	H	OH
erythrosine	H	H	H	I	O	I	OH

**Table 4:** Physicochemical properties of fluorescein derivatives approved for use in humans.

	Φ_F_	ex/em nm pH 7.4	anion : dianion : trianion pH 7.4	dye MW	clog P	clogD pH7.4	Uses
fluorescein [28,29]	0.80	494/521	61:39:0	332	3.0	−0.5	Intravital eye stain
eosin Y [29]	0.20	525/545	1:99:0	648	6.2	0.9	H&E stain
eosin B							Cell stain
carboxyfluorescein [30]	0.91	492/517	0:61:39	376	2.7	−4.0	Membrane impermeant
erythrosine [28,29]	0.02	521/534	1:99:0	880	6.8	1.8	Food colour, dental, radiopaque

**Table 5:** R-groups within a series of fluorescein derivatives not approved for use in humans.

Name	R2	R5	R6	R8=R13	R9	R10=R11	R12
fluorescein	H	H	H	H	O	H	OH
fluorescein butyl ester	(CH_2_)_3_CH_3_	H	H	H	O	H	OH
fluorescein butyl ether	H	H	H	H	O	H	O(CH_2_)_3_CH_3_
anthofluorescein	H	H	H	H	O	H	C_6_H_4_OH
Zinpyr-1	H	H	H	Cl	O	bipyridyl	OH

**Table 6:** Physicochemical properties of fluorescein derivatives not approved for use in humans.

	Φ_F_	ex/em nm pH 7.4	anion : dianion : trianion pH 7.4	dye MW	clog P	clogD pH7.4	Uses
fluorescein butyl ester [27]	0.80	455/525	90:0:0	388	4.7	3.7	rare
fluorescein butyl ether [27]	0.39	455/525	100:0:0	388	4.5	1.2	rare
anthofluorescein [31]	0.33	478/538	99:1:0	408	4.7	1.3	rare
Zn-Zinpyr-1 complex [32]	0.87	507/525	1:99:0	824	1.3	1.0	*in vivo* zinc

To observe sufficient small molecule fluorescence in tissue, the physicochemical properties of the molecule must enable sufficient tissue penetration. For example, molecular weight (MW) and log D influence the steady state flux of a molecule across biological barriers including skin [34] with ideal MW < 500 Da and pH 7 log D ~3. Log D is the pH dependent octanol/water distribution coefficient of all forms of an ionisable molecule.

Using intravital FLIM, our research group has identified a distinct signal for the fluorescence lifetimes of fluorescein (τ 4.1 ns) which is resolved from endogenous tissue fluorescence (τ < 2.7 and > 4.3 ns, see Table 1) in rat liver [10]. Subsequently, we have shown that the signals for the metabolite fluorescein glucuronide (τ 2.3 ns) and fluorescein (τ 3.4 ns) in the bile duct can be resolved using FLIM detection [35] and that protein binding lowers the fluorescence lifetime of fluorescein by 0.4 ns. We have also used intravital multiphoton microscopy to monitor rhodamine 123 and transport changes induced by the P-glycoprotein inhibitor cyclosporine A [36]. 

**Figure 2:**
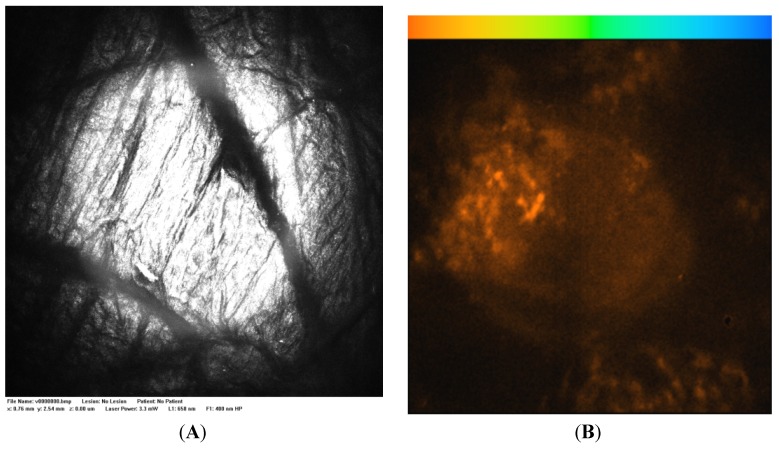
(**A**) Vivascope 658 nm reflectance image and (**B**) control fluorescence lifetime imaging (FLIM) image acquired 10 µm below the excised skin surface 3 h after application of phosphate buffered saline (PBS), magnification 400×; Field of view: 500 micron × 500 micron, FLIM color range 0–4 ns orange-blue.

**Figure 3:**
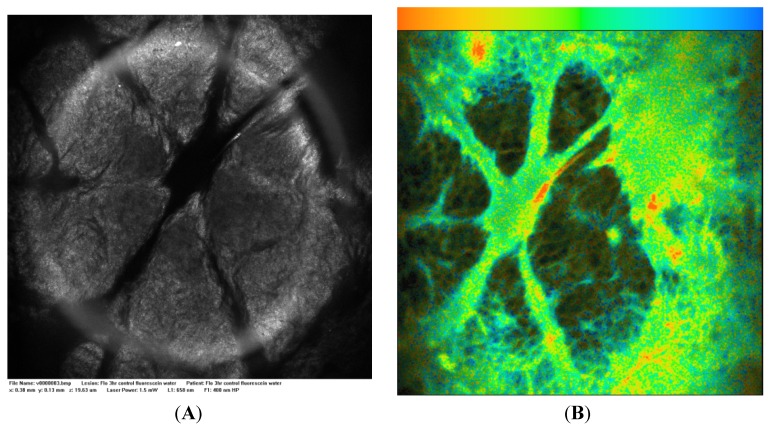
(**A**) Vivascope 658 nm reflectance image and (**B**) FLIM image acquired 20 µm below the skin surface 3 h after application of fluorescein in PBS at 16 mg/mL, magnification 400×; Field of view: 500 micron × 500 micron, FLIM color range 2–4 ns orange-blue.

**Figure 4:**
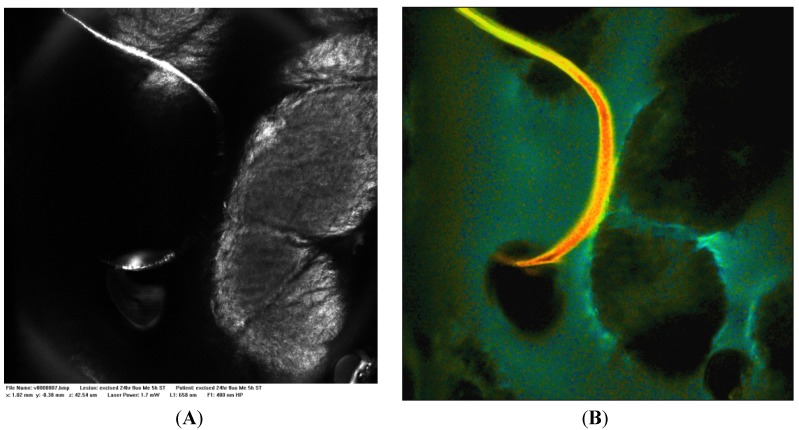
(**A**) Vivascope 658 nm reflectance image and (**B**) 488 nm FLIM image acquired 40 µm below the skin surface 5 h after application of fluorescein in microemulsion at 16 mg/mL, magnification 400×; Field of view: 500 micron × 500 micron, FLIM color range 2–4.5 ns orange-blue. Microemulsion system 2 (Tween 80 & Span 20), as reported in [33].

Figure 2A is a control Vivascope reflectance confocal microscope image of excised skin to which isotonic phosphate buffered saline (PBS) has been applied. Figure 2B is a FLIM image of the same area in which the endogenous signal has a low fluorescence lifetime (τ 0–0.5 ns, brown-orange) and low intensity compared to a stained sample. Figure 3A is a Vivascope reflectance confocal microscope image of excised skin to which a fluorescein solution was applied for three hours. The reflectance images highlight the dermatoglyphs or folds at the skin surface. Figure 3B is a FLIM image of the same area and together the two images illustrate that the fluorescein solution is retained at the surface with some binding to the surface features including the epidermis (τ 3.0 ns, green) and hairs (τ 2.5 ns, orange-yellow) as a line near the image centre. Binding at the stratum corneum layer of the epidermis highlights the detailed morphology of the surface tissue. Franz cell studies with excised epidermis have confirmed that microemulsions containing small molecules like fluorescein penetrate skin more rapidly with a hundred-fold higher steady-state flux (*J_ss_* μg/cm^2^.h) than aqueous solutions (e.g., microemulsion system 2 in [37]). Shown in Figure 4B, when fluorescein in this microemulsion was applied to excised, full thickness skin, the fluorescence intensity remained high at the surface and tissue staining was relatively weak. 

Fluorescein (20 mg/kg) has been has been used in surgical oncology for glioblastoma multiforme surgery [38]. Folate conjugated with fluorescein (folate-FITC) results in intra-operative, tumor-specific fluorescence and has been studied in ovarian cancer surgery using a custom built camera [39]. As expression of folate receptor-α (FR-α) is increased in 90–95% of epithelial ovarian cancer patients, folate-FITC specifically stains the tumours in most patients.

## 7. Rhodamine

The spectroscopic properties of the rhodamine series have recently been reviewed [40,41]. As shown in Table 7, Table 8, the members of the rhodamine series mostly have high Φ_F_ values and consequently are all useful fluorescence stains. 

Rigid polycyclic derivatives are in common use, such as the octacycle rhodamine 101 (Φ_F_ 0.9 [40]) and Texas red (a sulfonyl chloride derivative of rhodamine 101), which is used to form fluorescent protein conjugates. Sulforhodamines B [42] (shown [42] in Figure 1), G and 101 represent a bissulfonate class of compounds in which the carbon-2 carboxylate has been replaced by a sulfonate group. Many commercial dyes from Molecular Probes are rhodamine derivatives including Alexa Fluor 488, 514, 532, 546, 568, 594 and 610. Commercially available macromolecular rhodamine B dextran (e.g., 20, 40, 70 kDa) and fluorescein isothiocyanate dextran (e.g., 10, 20, 40 kDa) conjugates are used in animal studies to identify vasculature *in vivo* [43].

**Table 7:** R-groups within a series of rhodamine derivatives.

Name	R2	R5	R6	R8=R13	R9	R10=R11	R12
rhodamine 123	CH_3_	H	H	H	^+^NH_2_.Cl^−^	H	NH_2_
rhodamine B	H	H	H	H	HN^+^(CH_2_CH_3_)_2_.Cl^−^	H	HN(CH_2_CH_3_)_2_
rhodamine 6G	CH_2_CH_3_	H	H	CH_3_	HN^+^(CH_2_CH_3_).Cl^−^	H	HNCH_2_CH_3_
rhodamine 110	H	H	H	H	^+^NH_2_.Cl^−^	H	NH_2_
rhodamine 19	H	H	H	CH_3_	HN^+^(CH_2_CH_3_).Cl^−^	H	HN(CH_2_CH_3_)_2_

**Table 8:** Physicochemical properties of rhodamine derivatives.

	Φ_F_	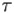 (ns)	ex/em nm pH 7.4	ionisation neutral: anion : dianion : trianion pH 7.4	dye MW	clog P	clogD pH7.4
rhodamine 123 [41]	0.90	3.6	511/534	1:0:0:0	344	2.9	2.4
rhodamine B [40,41]	0.53	1.9	553/572	1:2:0:0	442	2.3	2.3
rhodamine 6G [41,44]	0.95	3.9	526/555	95:0:0:0	444	5.4	5.3
rhodamine 110	-	-	500/525	4:1:0:0	330	−0.1	−0.2
rhodamine 101 [41]	0.96	4.1	560/589	1:0:0:0	491	2.6	2.6
rhodamine 19 [41]	0.95	4.2	535/546 [45]	9:91:0:0	415	1.1	1.5

## 8. Clinical and Preclinical Applications

### 8.1. Fluorescence Endomicroscopy

Translational optical imaging has been recently reviewed with emphasis on the available techniques and instruments [46]. A variety of instruments utilise endomicroscopy for the diagnosis of gastrointestinal diseases [9]. The Optiscan confocal fluorescence endoscope utilises a visible laser (488 nm) to generate a fluorescence signal and may be combined as contrast agents with fluorophores classified by the regulators as generally regarded as safe (GRAS e.g. acriflavine, fluorescein) [47]. A method involving intravenous injection of sodium fluorescein before confocal imaging with the Optiscan FIVE 1 has been reported for the diagnosis of brain tumors intraoperatively and identification of tumor margins during resection [48]. Intraepidermal injection of fluorescein followed by imaging with the Optiscan has been used to monitor progression of actinic keratoses (AK) and basal cell carcinoma (BCC) [49]. Acridine orange has been used to identify bowel inflammation in mouse colon [50] with a homebuilt confocal fluorescence microscope.

### 8.2. Dermatology

VivaScope 1500 Multilaser (Caliber Imaging & Diagnostics Rochester, NY, USA) includes fluorescence detection with three excitation wavelengths (488, 658, 785 nm). For example, intradermal injection of indocyanine green (ICG) enables assessment of epidermal morphology [51]. The DermaInspect (Jenlab, Jena, Germany) is a multiphoton tomograph instrument that utilises nIR light to produce visible fluorescence. Both instruments have been used for the diagnosis of skin disease including basal cell carcinoma

## 9. Advanced Imaging Techniques & Use of FLIM

FLIM is potentially useful for the simultaneous analysis of multiple fluorophores including endogenous molecules. By using a pulsed laser to measure the time taken for a fluorescence emission to occur, FLIM detection can distinguish between fluorophores with different fluorescence lifetimes. FLIM has recently been used to directly analyse H&E stained samples [24,52,53]. Eosin fluorescence dominates the FLIM signal (τ_ 2_~1100 ps) and is associated with protein staining of extracellular and intracellular protein [52,53]. As hematoxylin is non-fluorescent, nuclei are poorly stained for FLIM detection using H&E stain. The absence of haematoxylin enables eosin to also stain cell nuclei. Formalin fixed, unstained samples retained the endogenous fluorescence associated with FAD and have been used to identify the morphological changes associated with human ovarian and murine breast cancer tissue.

## 10. Formulation

Intravital fluorescent imaging applications commonly involve either injection of the dye in buffer or topical application of a buffered solution at the site of interest. Such simple solutions often cause limited dye penetration into tissue, which results in planar imaging of the tissue surface. Improved formulation methods provide the option to increase staining of sub-surface tissue with dyes including fluorescein using microemulsion [54], liposomes [55] or novel carriers such as chitosan [56]. 

## 11. Conclusion

The fluorescent dyes in widespread clinical use as injected imaging agents are limited to fluorescein, methylene blue, 5ALA and indocyanine green. Additional dyes permitted in topical applications include acridine orange, acriflavine and a few edible fluorophores including hypericin and curcumin. Endogenous fluorescence eliminates the need for a contrast agent and with advanced detectors or post-imaging analysis enables visualisation of subtle changes in tissue fluorescence. Advanced imaging techniques have led to an increasing number of applications in the diagnosis of diseases with fluorescence microscopy.
